# Sonographer quality management

**DOI:** 10.1007/s12574-019-00430-3

**Published:** 2019-06-12

**Authors:** Mieko Marriner

**Affiliations:** John Muir Medical Center, 2540 East Street, Concord, CA 94520 USA

**Keywords:** Sonographer registry system, ASE guideline and standard, Intersocietal Accreditation Commission (IAC), IAC standards and guideline for adult echocardiography accreditation, Quality improvement

## Abstract

**Background:**

The echocardiogram is the second most used diagnostic tool for cardiovascular patient care. Qualified sonographers are needed to consistently produce high-quality echocardiograms to provide high-quality patient care.

**Methods:**

Our department uses the three major management tools to assure sonographers are qualified. (1) ASE guidelines and standards. (2) Sonographer registry system and (3) quality improvement (QI) program with the accreditation of Echo Lab. QI measures are done quarterly assessing the appropriate use criteria for echocardiography, interpreter and sonographer variability, timeliness and completeness and correlations. The variabilities are assessed along ASE guidelines and standards.

**Results:**

QI measures are mainly done by Medical Director and Technical Director. Medical Director and Technical Director discuss with individual interpreter and sonographer after QI measures are done each quarter as the feedback. Through the feedback improving the individual sonographer skills and understanding of Echo result. Our Echo Lab is accredited by Intersocietal Accreditation Commission (IAC). Accredited Echo Labs have to follow the IAC standards for echocardiography which includes those QI measures. Every sonographer in our lab is registered and complied for CME requirement to update their knowledge and skills. Twice a year QI meeting will be held and discuss about those QI measures and make consensus as Echo Lab.

**Conclusions:**

Registered sonographer with systematic quality checking system of their work will be achieved improving the high-quality echocardiogram and leading to the high-quality patient care.

The echocardiogram is the second most used diagnostic tool following the electrocardiogram for cardiovascular patient care. High-quality cardiac images and hemodynamic assessments are dependent on the individual sonographer skills. To achieve high-quality echocardiograms in our lab, we manage the sonographer skills with three major components:

1. ASE guideline and standard [1–4].
2. Sonographer registry system.
3. Quality improvement (QI) program with the accreditation of Echo Lab.

Our Echo Lab follows the ASE guideline and standard for echocardiogram exams. Seven of the 24 × 30 in. sized ASE guideline posters are on the walls of the sonographer work stations as reference for both qualitative and quantitative echo preliminary report creation for the reading cardiologist.

11.5 × 17 in. flip chart posters of the ASE guidelines are also put on each of the desks in the cardiologist reading room.

Not all of the cardiologists or sonographers join the ASE. When new or updated guidelines are released by ASE, the Medical Director explains the guideline or update to every member at our regular non-invasive cardiology education classes. The class also features education regarding new Echo related medical procedures and accepts sonographer’s requests for education topics. When this education class is held at our campus, it is broadcast to our sister campus, and vice versa.

There are two sonographer registry bodies in USA. One is the American Registry of Diagnostic Medical Sonographers (ARDMS) and the other is Cardiovascular Credentialing International (CCI). Both organizations check a sonographer’s skill and knowledge of Echocardiography and related equipment.

Both ARDMS and CCI ask applicants to take a rigorous exam to demonstrate their knowledge and abilities in ultrasound physics and their particular speciality. ARDMS awards the Registered Diagnostic Cardiac Sonographer designation (RDCS) and CCI awards the Registered Cardiac Sonographer (RCS) designation to successful candidates. ARDMS requires 30 h of continuing medical education every 3 years and CCI requires 36 h every 3 years. Every sonographer in our Echo Lab is registered as RDCS or RCS or both and complies for CME requirement to update their knowledge and skills.

In addition to sonographers’ credentialing, Echo Labs can also receive accreditation from the Intersocietal Accreditation Commission (IAC). The goal of the IAC is to promote and ensure high-quality patient care [5]. The IAC sets standards, guidelines, procedures and documentation requirements. Each lab must demonstrate compliance with those requirements to be accredited. Our Echo Lab is accredited by IAC and comply requirements to reaccredit for every 3 years.

Among the many requirements is the implementation of a QI program. The measures provided by IAC for a QI program are [5]:

- Test appropriateness.
- Technical quality and, if applicable, safety of the imaging.
- Interpretive quality review.
- Report completeness and timeliness.
- Correlation.

*Test appropriateness* QI review and documentation are performed quarterly. The Technical Director selects thirty consecutive TTE, TEE, and stress echo studies and reviews each modality for appropriateness. The Technical Director assigns a score using the appropriate use criteria for echocardiography (AUC) [6].

In addition to this appropriateness score each of the thirty studies is reviewed for timeliness, which includes the procedure date, interpreted date, and signed date. Routine inpatient echo needs to be interpreted within 24 h of the completion of the examination. Outpatient studies must be interpreted by the end of the next business day. The final verified (by the interpreting physician) signed report must be completed within 48 h after interpretation [5].

All of the information for the thirty studies is recorded in spreadsheet form which calculates a percentage showing how many studies achieved the appropriateness and timeliness goals.

*Technical quality* The Technical Director checks the other sonographers technical quality by randomly selecting two studies in each modality and reviewing them for image quality, completeness of the study, and adherence to facility protocols. IAC offers a sample score sheet with a score of 3 meeting all goals, 2 indicates some deficiency and 1 is unacceptable.

The medical director checks the Technical Director’s studies using the same scoring method.

Five criteria are used to complete the checks [5]:

- Completeness (all required views, measurements and Doppler evaluations).
- Followed protocol.
- Image quality.
- Doppler quality.
- Measurements/accuracy.

The Technical Director reviews the results with each sonographer and discusses areas for improvement.

*Interpretive quality* The Technical Director randomly selects two interpretations in each modality for each physician to be reviewed.

The Medical director reads the sonogram without reviewing the original doctor’s interpretation. The Technical Director compares the Medical Director’s interpretation to the original doctor’s interpretation. When variability exists, the physicians must review the sonogram and agree as to the interpretation. Uniform sets of diagnostic criteria must also be discussed and agreed upon [5].

*Report completeness and timeliness* Ten random reports per quarter of all modalities performed in the facility (TTE, TEE, SE must be represented) are analyzed for [5]:

- Date of study.
- Date of Reading and finalized report.
- Completeness—(Did the report contain all required comments, measurement, demographics?).
- If discrepancies exists, the Medical Director must discuss with the interpretive physician.

*Correlation* The Technical Director quarterly selects two cases of each modality and performs the correlation analysis following the IAC guidelines. The Technical Director should note the discrepancies and document actions taken.

- *Correlation of TTE and confirmation of results* For those patients who have undergone TTE and other diagnostic procedures (such as cardiac catheterization, coronary angiograms or nuclear perfusion studies) or surgical intervention the results of TTE and other procedures must be routinely compared with regard to valvular abnormalities and left ventricular function [5].
- *Correlation of TEE and confirmation of results* For those patients have undergone TEE and surgical repair or other diagnostic procedures (such as coronary angiograms or nuclear perfusion studies), the results of TEE and other procedures should be routinely compared with regard to valvular abnormalities, left ventricular function and abnormalities of the aorta [5].
- *Correlation of SE and confirmation of results* For those patients who have undergone SE and other diagnostic procedures (such as coronary angiograms or nuclear perfusion studies), the results, of the SE and the other procedures should be routinely compared [5].

In addition to the five parts of the IAC’s QI process, a peer review system can be established. The Technical Director can randomly select one study. Copies of the study with patient and sonographer’s name redacted are scored by each sonographer.

Lastly, QI meetings can be held twice a year [5]. The results of all the reviews and checks are presented for analysis and discussion and try achieving the goal for improving the high-quality echocardiography and leading to the high-quality patient care.

Registered cardiac sonographers are tested for their skill and knowledge and must complete continuing education to maintain and update their skills and knowledge.

Systematic quarterly quality checks of the sonographer include their work completeness, following protocols and ASE guidelines, optimizing image and Doppler quality with the use of echo contrast, alignment of flow jet with ultrasound beam, gain control, and accuracy of measurements. These quarterly checks improve the sonographer’s study qualify thus improving the Echo Lab quality.

High-quality echocardiograms help doctors make decisions regarding patient treatment including when and how to intervene.

I believe high-quality echocardiograms lead to high-quality patient care.
